# Long-Term Outcomes After Laparoscopic vs Open Adhesiolysis for Small Bowel Obstruction: The LASSO Randomized Clinical Trial

**DOI:** 10.1001/jamasurg.2025.6726

**Published:** 2026-02-18

**Authors:** Panu Räty, Panu Mentula, Eija Haukijärvi, Risto Juusela, Heidi Wikström, Vesa Koivukangas, Berndt Enholm, Salomone Di Saverio, Arianna Birindelli, Fausto Catena, Ari Leppäniemi, Ville Sallinen

**Affiliations:** 1 Department of Abdominal Surgery, University of Helsinki and Helsinki University Hospital, Helsinki, Finland; 2 Tampere University Hospital, Tampere, Finland; 3 Vaasa Central Hospital, Vaasa, Finland; 4 Peijas Hospital, Vantaa, Finland; 5 Department of Surgery, Oulu University Hospital, Oulu, Finland; 6 Department of Surgery, Päijät-Häme Central Hospital, Lahti, Finland; 7 Maggiore Hospital Bologna, Bologna, Italy; 8 Department of Surgery, University of Bologna, Bologna, Italy; 9 University of Bologna, Bufalini Hospital Cesena, Italy; 10 Department of Transplantation and Liver Surgery, University of Helsinki and Helsinki University Hospital, Helsinki, Finland

## Abstract

**Importance:**

A laparoscopic approach for adhesive small bowel obstruction (SBO) requiring surgery provides quicker recovery compared with open surgery, but benefits in long-term follow-up are unclear.

**Objective:**

To compare SBO recurrence, incisional hernia, and quality of life (QOL) outcomes in long-term follow-up after laparoscopic vs open surgery.

**Design, Setting, and Participants:**

The Laparoscopic vs Open Adhesiolysis for Adhesive Small Bowel Obstruction (LASSO) randomized clinical trial was conducted in 8 hospitals in Finland and Italy between July 2013 and April 2018. In this study, the 5-year follow-up is reported. This was an international, multicenter, parallel, open-label randomized clinical trial including patients with clinical and radiological signs of adhesive SBO not resolving by conservative means. Study data were analyzed from February to May 2025.

**Interventions:**

Open vs laparoscopic adhesiolysis.

**Main Outcomes and Measures:**

SBO recurrence rate, incisional hernia incidence and QOL (Gastrointestinal Quality Of Life Index [GIQLI] and 36-item Short-Form Health Survey [SF-36]) within 5 years are reported using modified intention-to-treat and post hoc per-protocol analyses.

**Results:**

A total of 104 patients were randomized, and 100 (mean [SD] age, 69.2 [15.7] years; 65 female [65%]) were included in the analyses (49 in open surgery, 51 in laparoscopic surgery). At 1 year, 1 patient (2.3%) in the open-surgery group had recurrent SBO vs 2 patients (4.5%) in the laparoscopy group (odds ratio [OR], 2.05; 95% CI, 0.18-23.44; *P* >.99). Within 5 years, 3 patients (9.7%) in the open-surgery group had at least 1 recurrent SBO vs 4 patients (12.5%) in the laparoscopy group (OR, 1.33; 95% CI, 0.27-6.51; *P* >.99). Incisional hernias were detected in 2 patients (6.1%) in the open-surgery group vs 2 patients (6.3%) in the laparoscopy group (OR, 1.03; 95% CI, 0.14-7.82; *P* >.99). At 5-year follow-up, median (IQR) SF-36 score was 73.2 (52.8-85.9) in the open-surgery group and 67.1 (42.6-76.7) in the laparoscopy group (*P* = .23), and median (IQR) GIQLI scores were 118 (95-136) in the open-surgery group and 119 (102-129) in the laparoscopy group (*P* = .54).

**Conclusions and Relevance:**

Results of this randomized clinical trial reveal that, although the laparoscopic approach to adhesive SBO has small short-term benefits, it was not superior to open surgery based on long-term follow-up.

**Trial Registration:**

ClinicalTrials.gov Identifier: NCT01867528

## Key Points

**Question:**

Does laparoscopic surgery for adhesive small bowel obstruction decrease small bowel obstruction recurrence rate or incidence of incisional hernias and improve the long-term quality of life compared with open surgery?

**Findings:**

In this prespecified analysis of the Laparoscopic vs Open Adhesiolysis for Adhesive Small Bowel Obstruction (LASSO) randomized clinical trial including 100 participants, laparoscopy was not found to be superior to open surgery in terms of small bowel obstruction recurrence rate, incisional hernia incidence, or quality of life in 5-year follow-up.

**Meaning:**

Results suggest that open surgery is still a good option for the treatment of adhesive small bowel obstruction as long-term outcomes are similar to those of the laparoscopic approach; laparoscopy is a treatment alternative option for highly select patients due to short-term benefits.

## Teaser

This prespecified analysis of the Laparoscopic vs Open Adhesiolysis for Adhesive Small Bowel Obstruction (LASSO) randomized clinical trial investigates if laparoscopic surgery for adhesive small bowel obstruction decreases small bowel obstruction recurrence rate or incidence of incisional hernias and improves the long-term quality of life compared with open surgery.

## Introduction

Small bowel obstruction (SBO) is a common surgical emergency.^1^ The majority of SBOs are caused by adhesions in the peritoneal cavity.^2^ Although the majority of adhesive SBOs can be managed nonoperatively, up to 40% require surgical treatment.^3^ Laparotomy has been the standard for operative treatment of adhesive SBO as it ensures a wide field of vision of the abdomen, permits easier complete assessment of the bowel, and facilitates safe adhesiolysis. Laparoscopy has become a standard of care in many surgical issues in both elective and emergency surgery and has been increasingly used also to treat SBO. Pooled analyses of nonrandomized studies^4,5,6,7^ have suggested reduction in mortality, morbidity, wound infections, and length of hospital stay with the use of a laparoscopic approach. However, there is lack of evidence regarding long-term outcomes of the laparoscopic approach in treatment of adhesive SBO and other emergency general surgery procedures. In a propensity-matched analysis of a retrospective series, the laparoscopic approach was even associated with higher incidence of recurrent SBO requiring surgery,^8^ whereas other retrospective analyses observed fewer SBO-associated readmissions or reoperations using the laparoscopic approach.^9^ In other emergency surgery operations, laparoscopic appendectomy is associated with fewer short-term and long-term bowel obstructions compared with an open approach.^10^ Another study^11^ found laparoscopic approach to be associated with fewer postoperative incisional hernias and better quality of life (QOL) metrics compared with open surgery while treating peptic ulcer.

The LASSO (Laparoscopic vs Open Adhesiolysis for Adhesive Small Bowel Obstruction) trial was the first and, to the best of our knowledge, only randomized clinical trial to compare laparoscopic adhesiolysis with an open approach as a treatment for adhesive SBO in terms of length of postoperative hospital stay and morbidity.^12^ Our study showed that in selected patients, laparoscopic adhesiolysis provided faster recovery while there was no difference in short-term complications. However, high-quality evidence of long-term effects of laparoscopy compared with conventional open approach is scarce. Here, we present the 5-year results of the LASSO trial regarding QOL, SBO recurrence, and incisional hernia incidence. The main hypothesis was that laparoscopic approach could result in better QOL and fewer incisional hernias and SBO recurrences due to smaller incisions and lesser tissue trauma.

## Methods

### Study Design

The LASSO trial was an international, multicenter, open-label, parallel group, randomized clinical trial comparing laparoscopic adhesiolysis with open adhesiolysis in patients with acute adhesive SBO that did not resolve nonoperatively. The trial was carried out in 5 university hospitals and 3 community hospitals in 2 countries: Finland and Italy. The trial was approved by the ethical committee of Helsinki University Hospital on May 21, 2013, and by the institutional review board at each center. Results of the 30-day follow-up have been published earlier, together with detailed information of methods.^12^ The full trial protocol and statistical analysis plan have also been published earlier (Supplement 1 and Supplement 2).^13^ This study followed the Consolidated Standards of Reporting Trials (CONSORT) reporting guidelines.

### Participants

Patients with clinical and radiological (computed tomography) signs of acute adhesive SBO that did not resolve by nonoperative treatment were eligible for inclusion. Patients with anesthesiologic contraindication, patients younger than 18 years or older than 95 years, pregnant patients, institutionalized patients, those who had been in the hospital more than 1 week before surgical consultation, and patients with earlier bariatric surgery or signs of strangulation were excluded. To enable the selection of patients most likely having a single adhesive band causing the obstruction, patients with the following conditions were also excluded: suspected peritoneal carcinosis, known wide adhesions, previous open surgery for endometriosis, previous generalized peritonitis, abdominal malignancy (or remission <10 years), previous radiotherapy of the abdominal region, 3 or more earlier open abdominal operations, suspicion of other source of obstruction than adhesions, recent abdominal operation (within 30 days), previous laparotomy for the aorta or iliac vessels, and Crohn disease. All patients gave written informed consent to participate in the trial. Race and ethnicity data are not collected in Finland in official registries or statistics, and thus were not collected in this study.

### Randomization

Patients were randomly allocated (1:1) to receive open or laparoscopic adhesiolysis. A block randomization with randomly varying block size (2, 4, or 6) was stratified according to each center. The allocated intervention was not blinded.

### Procedures

Fluid balance and electrolyte disturbances were corrected preoperatively, and prophylactic antibiotics were administered just before incision. For open surgery, a midline incision was used, adhesions were dissected, and fascia and skin were then closed. For the laparoscopic approach, a standardized method (ie, entry using the open or optic port method, with port locations and count according to the surgeons’ discretion, bowel examination starting from the terminal ileum until the transition was identified, the obstructive adhesion was divided, and finally, fascial holes larger than 5 mm in size were closed) was used.^13^ All surgeons performing the procedures were required to have solid experience, with individual assessment by the authors, for complex laparoscopic procedures and needed to have performed at least 2 laparoscopic adhesiolysis operations before operating on trial patients.

Prespecified criteria for conversion to open surgery were used:

- Confirmed or suspected small bowel perforation, which is not amenable to laparoscopic suturing
- Transition site not identified
- Cause for obstruction not found
- Peritoneal carcinosis detected
- Presence of widespread diffuse adhesions
- Need for bowel resection (conversion could be made to mini-laparotomy to exteriorize the small bowel section requiring resection)

### Outcomes

The primary outcome of the LASSO trial was postoperative length of hospital stay and has been reported along with secondary outcomes assessable within 30 days.^12^ The long-term outcomes of 1- and 5-year follow-ups are reported here. The 1- and 5-year follow-up questionnaires were sent to patients by mail. If the patients did not respond, they were contacted by phone call. The occurrence of incisional hernias and possible treatments of these as well as incidents of new bowel obstructions were assessed. These were also assessed from the patients’ medical records, and all incisional hernias diagnosed by a physician were included. After a trial protocol change on October 2, 2018, the 5-year questionnaire also included the QOL questionnaires Gastrointestinal Quality of Life Index (GIQLI) and the 36-item Short Form Health Survey (SF-36).

### Statistical Analysis

Based on sample size calculations for the primary outcome,^12,13^ the study aimed to recruit 102 patients. Continuous outcomes (QIGLI and SF-36 scores) were not normally distributed and were analyzed using Mann-Whitney *U* test (after multiple imputation with median p-rule method^14,15^) and effect size was reported as r = Z / (N)^1/2^. Multiple imputation (linear regression method) with 10 imputed datasets was used only in GIQLI questionnaire analysis, if at least 75% of the questionnaire was filled out. The missing data were assumed to be missing at random. Categorical outcomes were analyzed using Fisher exact test and χ^2^ test and the effect size was reported as odds ratio (OR) with 95% CI. All analyses were performed with SPSS, version 29 (IBM Corp). *P* values were 2-sided, and *P* <.05 was considered statistically significant.

The main analyses were carried out according to the intention-to-treat principle. In addition, post hoc per-protocol analyses were carried out where patients who were randomized to laparoscopic adhesiolysis but underwent conversion to open surgery were excluded from analyses. Potential effects of attrition were examined by comparing original baseline demographics of patients who responded to 1-year follow up, 5-year QOL questionnaire, and patients who were randomized to initial study.^12^ Study data were analyzed from February to May 2025.

## Results

**Figure 1.**
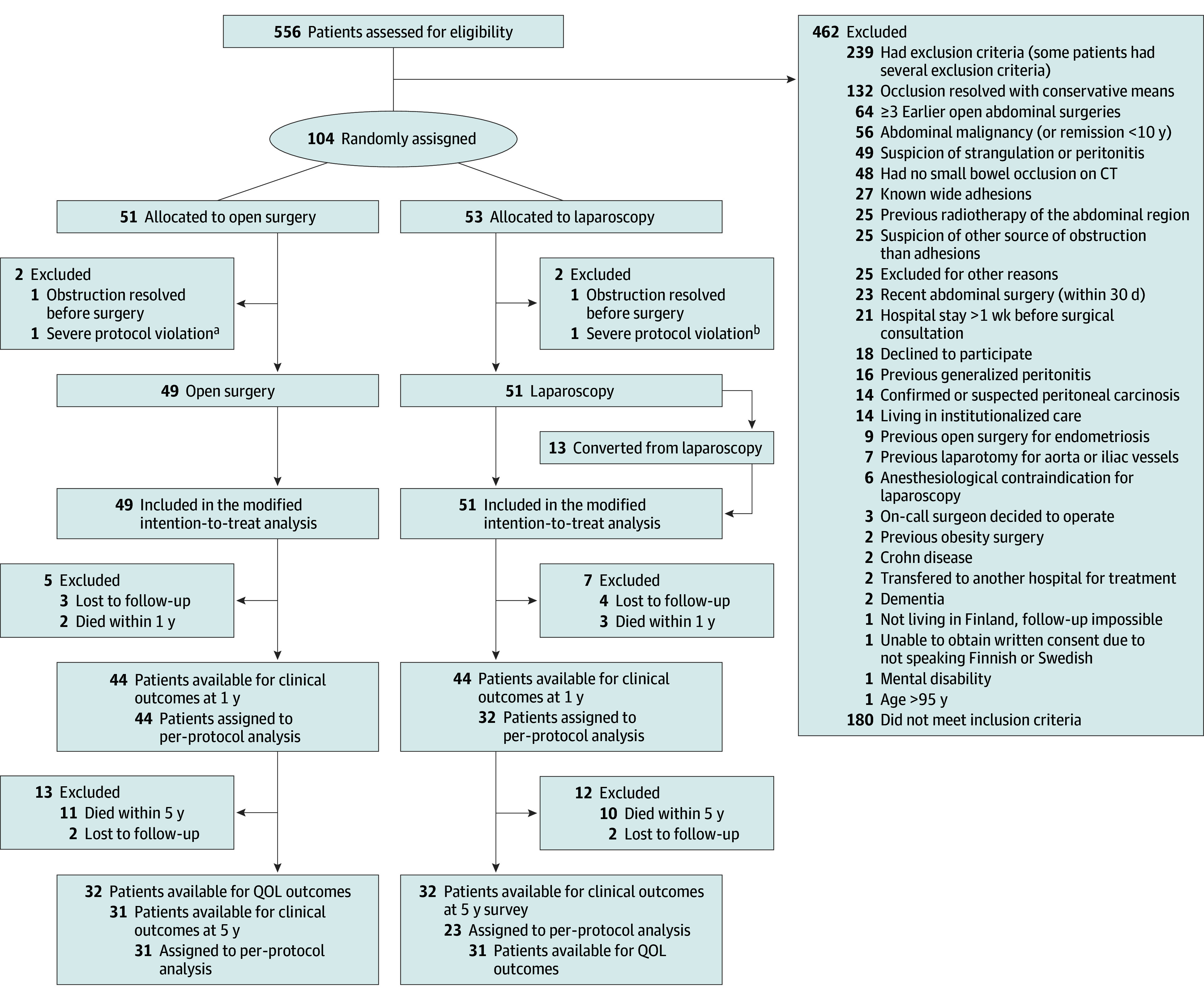
Study Flowchart CT indicates computed tomography; QOL, quality of life. ^a^Patient was randomly assigned despite meeting exclusion criteria (had earlier abdominal malignancy and radiotherapy). ^b^Patient was randomly assigned despite meeting exclusion criteria (earlier abdominal malignancy).

A total of 566 patients were assessed for eligibility, of whom 104 patients were recruited. Two patients (1 from each group) were excluded because the obstruction resolved before surgery, and 2 patients (1 from each group) were excluded because they were randomly assigned despite meeting the exclusion criteria. This resulted in 100 patients (mean [SD] age, 69.2 [15.7] years; 65 female [65%]; 35 male [35%]) being included in the modified intention-to-treat analyses (49 in open surgery, 51 in laparoscopic surgery). Basic characteristics of the patients included in the trial can be found from the original report.^12^ The flowchart of the patients is presented in Figure 1.

### One-Year Follow-Up Intention-to-Treat Analyses

Five patients (2 in the open-surgery group and 3 in the laparoscopy group) died within 1 year of recruitment. Of the remaining 95 patients who were alive at 1 year, 88 (93%) answered the 1-year follow-up survey. One patient (2.3%) in the open surgery group had a recurrent SBO episode (treated nonoperatively) vs 2 patients (4.5%) in the laparoscopy group (both patients were treated nonoperatively, the other patient in the open-surgery group had 2 episodes) (OR, 2.05; 95% CI, 0.18-23.44; *P* >.99) (Table 1). Incisional hernias were detected in 2 patients (4.5%) in the open-surgery group vs 2 patients (4.5%) in the laparoscopy group, of whom none had undergone operative repair (OR, 1.00; 95% CI, 0.14-7.43; *P* >.99) (Table 1). The basic demographics of the patients included in the analyses for 1-year outcomes were similar compared with the initial study group (eTable 1 in Supplement 3).

**Table 1.** Intention-to-Treat and Per-Protocol Analyses at 1 Year

Analysis type	Open surgery	Laparoscopy	OR (95% CI)	*P* value
**Intention to treat**				
No.	44	44	NA	NA
SBO within 1 y, No. (%)	1 (2)	2 (5)	2.05 (0.18-23.44)	>.99^a^
Nonoperatively treated SBO, No. (%)	1 (2)	2 (5)	2.05 (0.18-23.44)	>.99^a^
Operatively treated SBO, No. (%)	0	0	NA	NA
Incisional hernia within 1 y, No. (%)	2 (5)	2 (5)	1.00 (0.14-7.43)	>.99^a^
**Per protocol** ^b^				
No.	44	32		
SBO within 1 y, No. (%)	1 (2)	2 (6)	2.87 (0.25-33.07)	.57^a^
Nonoperatively treated SBO within 1 y, No. (%)	1 (2)	2 (6)	2.87 (0.25-33.07)	.57^a^
Operatively treated SBO within 1 y, No. (%)	0	0	NA	NA
Incisional hernia within 1 year, No. (%)	2 (5)	0	NA	.51^a^

Abbreviations: NA, not applicable; OR, odds ratio; SBO, small bowel obstruction.

^a^ Fisher exact test.

^b^ In the per-protocol analysis, patients who were randomized to laparoscopic adhesiolysis but underwent conversion to open surgery are excluded.

### One-Year Follow-Up Per-Protocol Analyses

In the per-protocol analyses, patients in the laparoscopy group who had undergone open surgery (those undergoing conversion during the initial operation were excluded) resulted in 32 patients for analyses at 1 year in the laparoscopy group. In the per-protocol analysis, 1 patient (2%) in the open-surgery group had a recurrent SBO episode (treated nonoperatively) vs 2 patients (6%) in the laparoscopy group (both treated nonoperatively) (OR, 2.87; 95% CI, 0.25-33.07; *P* = .57) (Table 1). In the per-protocol analysis, incisional hernias were detected in 2 patients (4.5%) in the open-surgery group vs no patients in the laparoscopy group (*P* = .51) (Table 1). Outcomes for patients randomized to the laparoscopic approach but converted to open surgery are shown in eTable 2 in Supplement 3. There were no differences in number of recurrent SBO episodes or incisional hernias between the laparoscopy group, open-surgery group, and conversion group within 1 year.

### Five-Year Follow-Up Intention-to-Treat Analyses

Of the total of 90 patients whose 5-year medical information was available (90%), 21 patients (11 in open surgery group and 10 in laparoscopy group) died within 5 years, and other medical information was available from 63 patients (31 in the open-surgery group and 32 in the laparoscopy group). Within 5 years, 3 patients (9.7%) in the open-surgery group had at least 1 recurrent SBO episode (1 patient treated only nonoperatively, 1 operatively, and 1 had 2 episodes; the first of these episodes was treated conservatively and the other operatively) vs 4 patients (13%) in the laparoscopy group (2 were treated only nonoperatively and 2 were treated first nonoperatively and then operatively) (OR, 1.33; 95% CI, 0.27-6.51; *P* >.99) (Table 2). Two incisional hernias (6.1%) were detected in the open-surgery group vs 2 patients (6.3%) in the laparoscopy group (OR, 1.03; 95% CI, 0.14-7.82; *P* >.99) (Table 2). The only operatively treated incisional hernia was in the laparoscopy group (in patients who were converted to open surgery).

**Table 2.** Intention-to-Treat and Per-Protocol Analyses at 5 Years

Analysis type	Open surgery	Laparoscopy	OR (95% CI)	*P* value
**Intention to treat**				
No.	31	32		
SBO within 5 y, No. (%)	3 (10)	4 (13)	1.33 (0.27-6.51)	>.99^a^
Nonoperatively treated SBO, No. (%)	2 (7)	4 (13)	2.07 (0.35-12.22)	.67^a^
Operatively treated SBO, No. (%)	2 (7)	2 (6)	0.97 (0.13-7.33)	>.99^a^
Incisional hernia within 5 y, No. (%)^b^	2 (6)	2 (6)	1.03 (0.14-7.82)	>.99^a^
**Per protocol** ^c^				
No.	31	23		
SBO within 5 y, No. (%)	3 (10)	3 (13)	1.40 (0.26-7.66)	>.99^a^
Nonoperatively treated SBO, No. (%)	2 (7)	3 (13)	2.18 (0.33-14.22)	.64^a^
Operatively treated SBO, No. (%)	2 (7)	1 (4)	0.66 (0.06-7.74)	>.99^a^
Incisional hernia within 5 y, No. (%)^b^	2 (6)	0	NA	.51^a^

Abbreviations: NA, not applicable; OR, odds ratio; SBO, small bowel obstruction.

^a^ Fisher exact test.

^b^ Open-surgery group n = 33.

^c^ In the per-protocol analysis, patients who were randomized to laparoscopic adhesiolysis but underwent conversion to open surgery are excluded.

### Five-Year Follow-Up Per-Protocol Analyses

In per-protocol analyses, patients in the laparoscopy group who had undergone open surgery (conversion during initial operation) were excluded, leaving 23 patients for analyses at 5 years in the laparoscopy group and 31 in the open-surgery group. In the per-protocol analysis, 3 patients (10%) in the open-surgery group had recurrent bowel obstruction episodes. One of these was treated nonoperatively, 1 operatively, and 1 had both operatively and nonoperatively treated SBOs. In the laparoscopy group, 3 patients (13%) had SBOs. Two of these were treated nonoperatively (1 patient 3 times and the other once) and 1 both nonoperatively and operatively (OR, 1.40; 95% CI, 0.26-7.66; *P* >.99) (Table 2). In the per-protocol analysis, incisional hernias were detected in 2 patients (6%) in the open-surgery group and in none in the laparoscopy group (*P* = .51) (Table 2). Outcomes for patients randomized to laparoscopy but converted to open surgery are shown in eTable 3 in Supplement 3. There were no differences in the number of recurrent SBO episodes or incisional hernias between the laparoscopy group, open-surgery group, and conversion group within 5 years.

### QOL at 5 Years

**Figure 2.**
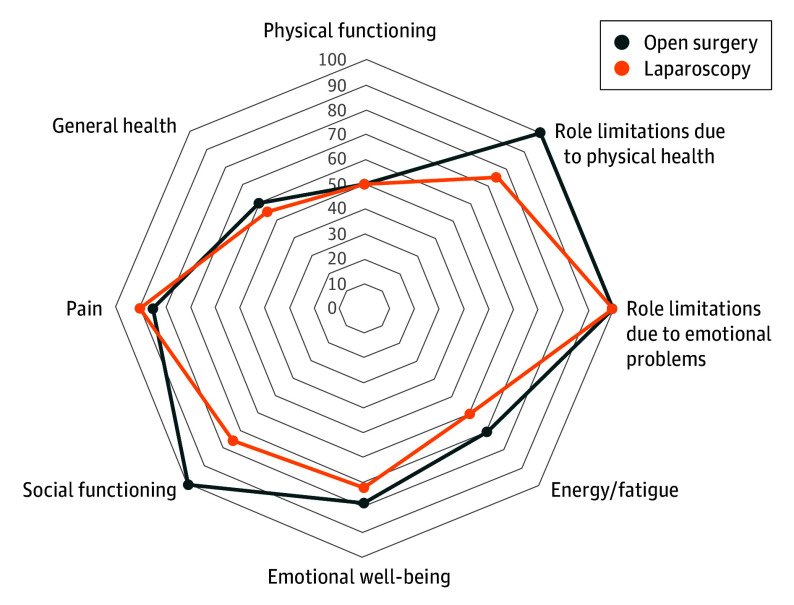
Radar Chart Analysis Showing the 36-Item Short-Form Health Survey (SF-36) Scales at 5-Year Follow-Up Higher value indicates better quality of life.

Of the 90 patients whose 5-year medical information was available, 63 patients (70%) responded to QOL questionnaires. Nineteen of the QIGLI questionnaires were incomplete (11 in the open-surgery group; 8 in the laparoscopy group) and were imputed and included in the analyses. Four of the SF-36 questionnaires were incomplete (2 in the open-surgery group; 2 in the laparoscopy group), and the questionnaires were analyzed by averaged items to form the scales. The median (IQR) SF-36 score was 73.2 (52.8-85.9) in the open-surgery group and 67.1 (42.6-76.7) in the laparoscopy group (*P* = .23). Item scores for both groups are shown separately in Table 3^16^ and visualized in Figure 2. The median (IQR) GIQLI scores were 118 (95-136) in the open-surgery group and 119 (102-129) in the laparoscopy group (*P* = .54) (Table 3^16^). There were no differences in baseline characteristics between the open and laparoscopy groups at 5-year follow-up in patients who responded to QOL questionnaires (eTable 4 in Supplement 3). However, compared with all patients included in the study, the patients who responded to the 5-year questionnaire were slightly younger (eTable 5 in Supplement 3).

**Table 3.** Intention-to-Treat Quality of Life Analysis at 5-Year Follow-Up

Analysis type	Median (IQR)		*P* value	Effect size^a^
	Open surgery	Laparoscopy		
SF-36, No.	26	29	NA	NA
GIQLI, No.	32	31	NA	NA
GIQLI	118 (95-136)	119 (102-129)	.54^b^	0.08
SF-36, average of scales	73.2 (52.8-85.9)	67.1 (42.6-76.7)	.23^c^	0.15
SF-36, physical functioning	50 (42.5-50.0)	50 (37.5-50.0)	.23^c^	0.16
SF-36, role limitations due to physical health	100 (18.8-100)	75 (0-100)	.34^c^	0.13
SF-36, role limitations due to emotional problems	100 (58.3-100)	100 (33.3-100)	.36^c^	0.12
SF-36, energy/fatigue	70 (50.0-81.3)	60 (45.0-75.0)	.14^c^	0.20
SF-36, emotional well-being	78 (66.0-92.0)	72 (62.0-85.0)	.25^c^	0.15
SF-36, social functioning	100 (62.5-100)	75 (62.5-100)	.12^c^	0.20
SF-36, pain	85 (57.5-100)	90 (55.0-100)	.98^c^	0.004
SF-36, general health	60 (47.5-71.3)	55 (40.0-67.5)	.35^c^	0.12

Abbreviations: GIQLI, Gastrointestinal Quality of Life Index; NA, not applicable; SF-36, 36-item Short Form Health Survey.

^a^ Effect size reported as r = Z / (N)^1/2^, where r <0.3 means small effect, r = 0.3-0.5 means medium effect, and r >0.5 means large effect.^16^

^b^ The GIQLI questionnaire’s *P* values are analyzed after multiple imputation with median *P* rule method.

^c^ Mann-Whitney *U* test.

## Discussion

In this long-term follow-up study of the LASSO randomized clinical trial comparing open surgery to a laparoscopic approach in adhesive SBO, laparoscopy was not found to be superior to open surgery in terms of SBO recurrence, incisional hernias, or QOL in 5-year follow-up. Although the study is limited by the small number of patients, higher numerical values of recurrent SBOs and incisional hernias were noted in the laparoscopy group, although no statistically significant differences were detected. These findings contradict the current evidence stemming from retrospective, uncontrolled series, suggesting that a laparoscopic approach would reduce the incidence of recurrent SBOs.

The evidence of laparoscopic treatment of SBO is limited. A recent systematic review and meta-analysis^17^ showed that laparoscopic adhesiolysis was associated with beneficial short-term effects. Cesena guidelines^18^ suggest careful step-by-step laparoscopic approach for SBO in stable patients although the evidence is based almost only on short-term results. The incidence of recurrent SBOs in our present study was relatively low: 3.4% at 1-year follow-up and 11.1% at 5-year follow-up. This can be partly explained by the reduced risk of recurrence after operative treatment of adhesive SBO.^19^ In other indications, laparoscopy is associated with decreased risk for long-term SBOs.^10^ This can be partly explained by lesser tissue trauma during surgery, which can decrease risk for new adhesions. However, in the present study, there were no statistically significant differences between SBO rates in the treatment groups, and the number of recurrent SBOs was slightly higher in the laparoscopy group. A retrospective study^8^ reported an increased incidence of recurrent SBOs associated with laparoscopic adhesiolysis, which can result from occult adhesions outside the obstructed site that are left untreated. In our present study, only the patients who had a high susceptibility for a single adhesive band were included. This decreases the likelihood of multiple adhesions.

One potential benefit of laparoscopic surgery is thought to be a decreased likelihood of incisional hernia formation compared with open surgery using a midline laparotomy incision.^20^ In our study, there were no differences in hernia formation between the randomization groups, but the study was underpowered to reveal small differences. A large nationwide cohort study^21^ found a small but significant difference in the incidence of incisional hernias after open vs laparoscopic surgery for colon cancer. In our study, the incidence of hernias was highest in the group where laparoscopic surgery was converted to open surgery, and there were no incisional hernias detected in patients who were operated on solely using the laparoscopic approach, but this needs to be interpreted with caution as numbers are small.

According to existing literature, the QOL after operative treatment for SBO has not been extensively studied. In our study, we examined QOL using 2 widely known questionnaires: GIQLI and SF-36. There were no differences in the QOL scores between the treatment groups.

Although the long-term outcomes did not differ between laparoscopy and open surgery, surgeons need to consider the economic burden of different procedures. Laparoscopic equipment is more expensive, and time in the operating room can be longer in laparoscopic procedures; however, faster recovery using the laparoscopic approach can reduce both direct (hospital) and indirect (sick leave) costs.^22^ In this trial, the time in the operating room did not differ between the study groups.^12^

### Strengths and Limitations

Our study has some strengths. One strength is the randomized setting and the rather good follow-up rate, although the patients who responded to QOL questionnaires at 5 years were slightly younger than those from the initial cohort. The response rate in the 1-year survey was sufficient 93%, but only 70% of patients filled out the 5-year survey and responded to QOL questionnaires.

There are several limitations to this study. First, the study group was rather small to study long-term results of 2 operative strategies, and this resulted in a low number of recurrent SBOs and incisional hernias. This can result in low statistical power to detect differences and may have led to uncertain estimates. Also, patient recruitment is difficult in the emergency surgery setting, and the present study required almost 5 years to recruit patients in 8 hospitals. The long recruitment period was also partly due to strict inclusion and exclusion criteria, which aimed to form a study cohort of selected patients who would be ideal candidates for the laparoscopic approach. Although we took into account the number of previous operations in our study patients, we did not specify the types of previous surgeries or incisions (or their length), which could have an effect on the amount of intra-abdominal adhesions. As in many randomized clinical trials, this trial has multiplicity by multiple subgroup analyses and repeated analyses in different (yet prespecified) time points. Thus, no strong inferences can be drawn from the post hoc per-protocol outcomes or outcomes of patients converted from laparoscopy to open surgery.

## Conclusions

This prespecified analysis of the LASSO randomized clinical trial highlights the fact that open surgery is still an option to manage SBO in patients, as long-term outcomes were not worse in open adhesiolysis compared with the laparoscopic approach. Laparoscopy has some benefits for slightly quicker short-term recovery and can thus still be considered in highly selected patients in whom a single adhesive band is anticipated.

## Notes

Supplement 1.
Trial Protocol.

Supplement 2.
Statistical Analysis Plan.

Supplement 3. **eTable 1.** Baseline Demographics of Patients Who Were Included to Initial Study and Patients Who Were in 1-Year Analysis **eTable 2.** One-Year Analysis of Outcomes for Patients Randomized to Laparoscopic Approach but Converted to Open Surgery **eTable 3.** Five-Year Analysis of Outcomes for Patients Randomized to Laparoscopic Approach but Converted to Open Surgery **eTable 4.** Baseline Demographics of Patients Who Responded to 5-Year QOL (Quality of Life) Questionnaire **eTable 5.** Baseline Demographics of Patients Who Were Included to Initial Study and Patients Who Were in 5-Year QOL Analysis

Supplement 4.
Data Sharing Statement.
