# Supplementary material for: Long-Term Outcomes After Laparoscopic vs Open Adhesiolysis for Small Bowel Obstruction: The LASSO Randomized Clinical Trial
Source: JAMA Surg. 2026 Feb 18;161(4):381–8. doi: 10.1001/jamasurg.2025.6726 (PMC12917750; doi:10.1001/jamasurg.2025.6726)
Supplement: Supplement 1. — Trial Protocol. [file jamasurg-e256726-s001.pdf]

Research protocol

Laparoscopic versus open adhesiolysis for small
bowel obstruction - a multicenter, prospective,
randomized, controlled trial

Ville Sallinen, Paulina Salminen, Eija Haukijärvi, Imre Ilves, Juha Saarnio, Vesa Koivukangas, Jyrki Kössi, Mikael Victorzon, Heidi Wikström, Ari Leppäniemi, Panu Mentula

Background

Bowel obstruction is one of the most common emergency surgical condition. Obstructive site is at 80% of the cases in small bowel. Adhesions are most common reason for small bowel obstruction (1,2). Most of the small bowel obstructions can be treated with non-operative treatment (3,4). Significant portion of the obstruction still need surgical intervention. Currently, the standard is to do explorative laparotomy. Commonly, the reason for obstruction is one adhesion and treatment is simple: to cut the band causing the adhesion. Laparoscopic approach seems ideal for such a procedure, preventing the morbidity of a laparotomy incision. First publications describing laparoscopic adhesiolysis in SBO are from the 1990's (5). Since then, several retrospective series comparing open and laparoscopic approach have been published. A recent meta-analysis pooled patients from four studies, including a total of 334 patients. Meta-analysis showed that patients treated by the laparoscopic approach had less complications, and faster return of bowel function (6). A recent consensus conference guideline recommends laparoscopic approach for selected patients (1). However, the scientific background of the recommendation is week, as there are no randomized or prospective studies of this subject. One of the drawbacks of laparoscopic approach is a concern for iatrogenic bowel perforation. In one report, the rate of bowel lesion in laparoscopic adhesiolysis was 6.6%, and only 84% were detected during the operation (7). In addition, there are no studies comparing the recurrence of small bowel obstructions between these two operative methods in long term follow-up.

Hypothesis

Laparoscopic adhesiolysis is suitable for treatment of acute small bowel obstruction and it shortens the hospital-stay time without increase in morbidity.

Methods

Centers

Helsinki university hospital

Turku university hospital
Tampere university hospital
Oulu university hospital
Kuopio university hospital
Päijät-Häme central hospital
Vaasa central hospital
Peijas hospital

Patient evaluation and selection

Inclusion criteria

– All patients with clinical and computed tomography-diagnosed adhesive small bowel obstruction
– Obstruction is not relieved by conservative methods (nasogastric tube, nil per os) including Gastrografin® is not passed to colon within 8 hours OR 48-hour conservative treatment without Gastrografin® if allergy to iodinine.

Exclusion criteria:

– Strong suspicion of strangulation or clinical peritonitis requiring an urgent operative intervention
– Previously confirmed or strongly suspected peritoneal carcinosis
– Previously confirmed wide diffuse adhesions of the abdominal cavity
– Previous open surgery for endometriosis
– Previous generalized peritonitis (not including local peritonitis such as appendicitis)
– Active abdominal malignancy or remission of less than 10 years' duration
– Previous radiotherapy of the abdominal region
– Previous obesity surgery
– 3 or more earlier open abdominal operations (not including caesarean section(s))
– Suspicion of other cause for obstruction than adhesions in CT-scan
– Recent abdominal operation (within 30 days)
– Previous laparotomy for aorta or iliac vessels
– Crohn's disease
– Anesthesiological contraindication for laparoscopy
– No informed consent
– Age less than 18 years or over 95 years
– Pregnancy
– Patient living in institutionalized care (such as health centre ward), not including retirement homes
– A hospital stay of more than one week prior to surgical consultation

Randomization procedure

Patients are randomly allocated (1:1) to either laparoscopic or open surgery. Randomization is done using block randomization with randomly varying block size (2–6) stratified by each study center. Cards with participants' randomization number and randomization group are sealed within numbered envelopes. Randomization and sealing within envelopes is done at the main research center (Helsinki) and letters are sent to each participating center at the beginning of the trial. The envelope is opened only after patient fulfills inclusion criteria, none of the exclusion criteria are met, and patient has

agreed to participate in the study and has given a written consent. Envelopes are opened in numerical order. Operation is scheduled after randomization.

Recommendation for non-operative treatment before randomization

At emergency department, the nasogastric tube is set and blood samples (complete blood count, electrolytes, creatinine, CRP, liver enzymes, lactate, d-dimer) and abdominal CT ordered. Abdominal CT is done with IV contrast if possible. PO contrast is not used. Gastrografin passage is done after abdominal CT, but earliest 12 hours after nasogastric tube insertion. If po contrast does not pass to colon in 8 hours and patient meets the inclusion criteria and has no exclusion criteria, the patient is asked for study participation.

Intervention

Pre- and perioperative treatment
Fluid balance and electrolyte disturbances are corrected. Prophylactic cefuroxime 1500 mg and metronidazole 500 mg are administered intravenously just before the incision. An epidural catheter may be placed if recommended by the anesthesiologist. A nasogastric tube is inserted.

Laparoscopic technique
The first port is inserted using open approach or by using an optic port. Subsequent ports are inserted under direct vision. The location of the ports is left to the surgeon's discretion. The abdominal cavity is inspected and the caecum located and identified. Laparoscopic forceps are used to examine the small bowel starting from the terminal ileum until the transition site is identified. Dilated small bowel loops are not grasped but can be mobilized by grasping the mesenterium. Once the transition site is identified, the obstructing adhesions are divided, and the bowel is inspected for vitality. Ports are removed under vision, and possible bleeding is primarily controlled by ligatures. The fascial holes of ports over 5 mm are closed. A nasogastric tube is left in place.

Open surgical technique
A midline incision is made, and the abdominal cavity is inspected. The small bowel is examined until the transition site is located. Adhesions causing obstruction are divided. Excess fluid within small bowel is pushed into the stomach and the stomach is emptied using a nasogastric tube. The fascia is closed using continuous or interrupted sutures at surgeon's discretion.

Postoperative treatment

The nasogastric tube is kept in place until the secretion is less than 500 ml per 8 hours. After the removal of the nasogastric tube, the patient can drink up to 200 ml per 6 hours. If no nausea develops, patient may drink freely. Proton pump inhibitors are used for the length of the hospital stay. Thrombosis prophylaxis is commenced 6 hours after surgery, if there is no suspicion of postoperative hemorrhage. Ibuprofen, paracetamol, tramadol, and oxycodone can be used for pain. Pain is evaluated using a visual analogue scale daily and before administering painkillers.

Criteria for discharge
– Passage of stool

– The patient tolerates per oral nutrition
– Sufficient pain relieve is achieved with ibuprofen, paracetamol, and/or tramadol.

Unresolving obstruction after surgery
If the obstruction if not resolved despite surgical treatment, the patient can undergo radiological imaging studies and/or surgical exploration (open or laparoscopic) at the discretion of the surgeon.

Follow-up
Patients of working age are given sick leave. The length of sick leave is at the discretion of treating physician, who is taking into consideration the patient's age and type of work (physical or desk job). A follow-up call is scheduled within 30 days, and return to work, possible late complications and readmissions are registered. Follow-up questionnaires are sent 1, 5 and 10 years after the randomization, and in case of no response, patients are contacted by telephone. Information about possible hernias and recurrent bowel obstructions are solicited.

Surgeons
The same surgeons perform both open and laparoscopic operations. All participating surgeons must have solid experience and skills of complex laparoscopic procedures, and need to have performed at least two laparoscopic adhesiolysis for small bowel obstruction before operating on patients participating in the trial.

Primary endpoint
– Length of post-operative hospital stay (days)

Secondary endpoints
– Passage of stool (post-operative days)
– Commencement of enteral nutrition (post-operative days)
– 30-day mortality
– Complications (all causes), graded by Clavien-Dindo classification
– Number of participants with iatrogenic small bowel lesions
– Number of participants with readmission(s)
– Number of participants with failure to resolve obstruction
– Pain scores on the Visual Analog Scale
– Length of epidural catheter analgesia (days)
– Total need of opioids in milligrams
– Length of sick leave (days)
– Conversion rate (laparoscopic group)

Tertiary endpoints
– Number of participants who develop ventral hernia
– Number of patients with recurrent adhesive small bowel obstruction

Collected parameters in Case Report Form.

Sample size calculation

We have analyzed laparoscopic (n=12) and open (n=16) small bowel adhesiolysis operations performed in Helsinki University hospital 2010-2012. Patients who were operated laparoscopically, the mean hospital treatment time was 4,75 days and by open surgery 9,87 days. The mean standard deviation of treatment times were 3,65 days in laparoscopy group and 7,16 days in open surgery group. We estimated the standard deviation to be 3.75 days in laparoscopic group and 5 days in open surgery group. Sample size is calculated to be able to demonstrate 2.5 days difference in the post-operative length of stay. 102 patients are needed to achieve 80% power with a significance level of 0.05.

Schedule

The study is planned to start in summer 2013. The interim analysis will be done after recruitment of 51 patients. The trial is continued if there is no statistically significant difference between treatment groups in primary endpoint. However, if it is estimated that statistically significant difference is not possible to achieve with 102 trial patients, the study is ended. In other situations, the trial will end after recruitment and randomization of 102 patients. It is estimated that the recruitment will take 5 years, and the follow-up period is until 2038.

Registration

This trial will be registered at ClinicalTrials.gov

Privacy

Patient identification details are collected to two separate files. In one file is collected all patients who have adhesive small bowel obstruction in CT scan. To other file is collected those patients who are recruited to the trial. The data is stored and analyzed without identification details. Patients get randomization number which is stored with identification details to separate file. The data is stored in locked space and electronic data is stored in Helsinki university hospital's computer which is protected by password.

References

1. Vettoretto N, Carrara A, Corradi A, De Vivo G, Lazzaro L, Ricciardelli L, et al. Laparoscopic adhesiolysis: consensus conference guidelines. Colorectal Disease. 2012 Mar 30;14(5):e208–15.

2. Barmparas G, Branco BC, Schnüriger B, Lam L, Inaba K, Demetriades D. The Incidence and Risk Factors of Post-Laparotomy Adhesive Small Bowel Obstruction. J Gastrointest Surg. 2010 Mar 30;14(10):1619–28.

3. Abbas S BIPB. Oral water soluble contrast for the management of adhesive small bowel obstruction. 2010 Jan 1;:1–24.

4. Branco BC, Barmparas G, Schnüriger B, Inaba K, Chan LS, Demetriades D. Systematic review and meta-analysis of the diagnostic and therapeutic role of water-soluble contrast agent in adhesive small bowel obstruction. Br J Surg. 2010 Mar 4;97(4):470–8.

5. Clotteau JE, Premont M. [Occlusion by adhesions treated by celioscopic section]. Presse Med. 1990 Jun 23;19(25):1196.

6. Li MZ, Lian L, Xiao L, Wu W, He Y, Song X. Laparoscopic versus open adhesiolysis in patients with adhesive small bowel obstruction: a systematic review and meta-analysis. AJS. Elsevier Inc; 2012 Jul 11;:1–8.

7. O'Connor DB, Winter DC. The role of laparoscopy in the management of acute small-bowel obstruction: a review of over 2,000 cases. Surg Endosc. 2011 Sep 5;26(1):12–7.

Research protocol version 1.1

Laparoscopic versus open adhesiolysis for small bowel obstruction - a multicenter, prospective, randomized, controlled trial

V1.1. 30.4.2014

Ville Sallinen, Paulina Salminen, Eija Haukijärvi, Imre Ilves, Juha Saarnio, Vesa Koivukangas, Jyrki Kössi, Mikael Victorzon, Heidi Wikström, Ari Leppäniemi, Panu Mentula

Background

Bowel obstruction is one of the most common emergency surgical condition. Obstructive site is at 80% of the cases in small bowel. Adhesions are most common reason for small bowel obstruction (1,2). Most of the small bowel obstructions can be treated with non-operative treatment (3,4). Significant portion of the obstruction still need surgical intervention. Currently, the standard is to do explorative laparotomy. Commonly, the reason for obstruction is one adhesion and treatment is simple: to cut the band causing the adhesion. Laparoscopic approach seems ideal for such a procedure, preventing the morbidity of a laparotomy incision. First publications describing laparoscopic adhesiolysis in SBO are from the 1990's (5). Since then, several retrospective series comparing open and laparoscopic approach have been published. A recent meta-analysis pooled patients from four studies, including a total of 334 patients. Meta-analysis showed that patients treated by the laparoscopic approach had less complications, and faster return of bowel function (6). A recent consensus conference guideline recommends laparoscopic approach for selected patients (1). However, the scientific background of the recommendation is week, as there are no randomized or prospective studies of this subject. One of the drawbacks of laparoscopic approach is a concern for iatrogenic bowel perforation. In one report, the rate of bowel lesion in laparoscopic adhesiolysis was 6.6%, and only 84% were detected during the operation (7). In addition, there are no studies comparing the recurrence of small bowel obstructions between these two operative methods in long term follow-up.

Hypothesis

Laparoscopic adhesiolysis is suitable for treatment of acute small bowel obstruction and it shortens the hospital-stay time without increase in morbidity.

Methods

Centers

Helsinki university hospital
Turku university hospital
Tampere university hospital
Oulu university hospital
Kuopio university hospital
Päijät-Häme central hospital
Vaasa central hospital
Peijas hospital

Patient evaluation and selection

Inclusion criteria

– All patients with clinical and computed tomography-diagnosed adhesive small bowel obstruction
– Obstruction is not relieved by conservative methods (nasogastric tube, nil per os) including Gastrografin® is not passed to colon within 8 hours (48-hour conservative treatment without Gastrografin® is allowed if Gastrografin® is contraindicated (e.g. allergy) or not available).

Exclusion criteria:

– Strong suspicion of strangulation or clinical peritonitis requiring an urgent operative intervention
– Previously confirmed or strongly suspected peritoneal carcinosis
– Previously confirmed wide diffuse adhesions of the abdominal cavity
– Previous open surgery for endometriosis
– Previous generalized peritonitis (not including local peritonitis such as appendicitis)
– Active abdominal malignancy or remission of less than 10 years' duration
– Previous radiotherapy of the abdominal region
– Previous obesity surgery
– 3 or more earlier open abdominal operations (not including caesarean section(s))
– Suspicion of other cause for obstruction than adhesions in CT-scan
– Recent abdominal operation (within 30 days)
– Previous laparotomy for aorta or iliac vessels
– Crohn's disease
– Anesthesiological contraindication for laparoscopy
– No informed consent
– Age less than 18 years or over 95 years
– Pregnancy
– Patient living in institutionalized care (such as health centre ward), not including retirement homes
– A hospital stay of more than one week prior to surgical consultation

Randomization procedure

Patients are randomly allocated (1:1) to either laparoscopic or open surgery. Randomization is done using block randomization with randomly varying block size

(2–6) stratified by each study center. Cards with participants' randomization number and randomization group are sealed within numbered envelopes. Randomization and sealing within envelopes is done at the main research center (Helsinki) and letters are sent to each participating center at the beginning of the trial. The envelope is opened only after patient fulfills inclusion criteria, none of the exclusion criteria are met, and patient has agreed to participate in the study and has given a written consent. Envelopes are opened in numerical order. Operation is scheduled after randomization.

Recommendation for non-operative treatment before randomization

At emergency department, the nasogastric tube is set and blood samples (complete blood count, electrolytes, creatinine, CRP, liver enzymes, lactate, d-dimer) and abdominal CT ordered. Abdominal CT is done with IV contrast if possible. PO contrast is not used. Gastrografin passage is done after abdominal CT, but earliest 12 hours after nasogastric tube insertion. If po contrast does not pass to colon in 8 hours and patient meets the inclusion criteria and has no exclusion criteria, the patient is asked for study participation.

Intervention

Pre- and perioperative treatment

Fluid balance and electrolyte disturbances are corrected. Prophylactic cefuroxime 1500 mg and metronidazole 500 mg are administered intravenously just before the incision. An epidural catheter may be placed if recommended by the anesthesiologist. A nasogastric tube is inserted.

Laparoscopic technique

The first port is inserted using open approach or by using an optic port. Subsequent ports are inserted under direct vision. The location of the ports is left to the surgeon's discretion. The abdominal cavity is inspected and the caecum located and identified. Laparoscopic forceps are used to examine the small bowel starting from the terminal ileum until the transition site is identified. Dilated small bowel loops are not grasped but can be mobilized by grasping the mesenterium. Once the transition site is identified, the obstructing adhesions are divided, and the bowel is inspected for vitality. Ports are removed under vision, and possible bleeding is primarily controlled by ligatures. The fascial holes of ports over 5 mm are closed. A nasogastric tube is left in place.

Open surgical technique

A midline incision is made, and the abdominal cavity is inspected. The small bowel is examined until the transition site is located. Adhesions causing obstruction are divided. Excess fluid within small bowel is pushed into the stomach and the stomach is emptied using a nasogastric tube. The fascia is closed using continuous or interrupted sutures at surgeon's discretion.

Postoperative treatment

The nasogastric tube is kept in place until the secretion is less than 500 ml per 8 hours. After the removal of the nasogastric tube, the patient can drink up to 200 ml per 6 hours. If no nausea develops, patient may drink freely. Proton pump inhibitors are used for the length of the hospital stay. Thrombosis prophylaxis is commenced 6 hours after surgery, if there is no suspicion of postoperative hemorrhage. Ibuprofen, paracetamol, tramadol, and

oxycodone can be used for pain. Pain is evaluated using a visual analogue scale daily and before administering painkillers.

Criteria for discharge
– Passage of stool
– The patient tolerates per oral nutrition
– Sufficient pain relieve is achieved with ibuprofen, paracetamol, and/or tramadol.

Unresolving obstruction after surgery
If the obstruction if not resolved despite surgical treatment, the patient can undergo radiological imaging studies and/or surgical exploration (open or laparoscopic) at the discretion of the surgeon.

Follow-up
Patients of working age are given sick leave. The length of sick leave is at the discretion of treating physician, who is taking into consideration the patient's age and type of work (physical or desk job). A follow-up call is scheduled within 30 days, and return to work, possible late complications and readmissions are registered. Follow-up questionnaires are sent 1, 5 and 10 years after the randomization, and in case of no response, patients are contacted by telephone. Information about possible hernias and recurrent bowel obstructions are solicited.

Surgeons
The same surgeons perform both open and laparoscopic operations. All participating surgeons must have solid experience and skills of complex laparoscopic procedures, and need to have performed at least two laparoscopic adhesiolysis for small bowel obstruction before operating on patients participating in the trial.

Primary endpoint
– Length of post-operative hospital stay (days)

Secondary endpoints
– Passage of stool (post-operative days)
– Commencement of enteral nutrition (post-operative days)
– 30-day mortality
– Complications (all causes), graded by Clavien-Dindo classification
– Number of participants with iatrogenic small bowel lesions
– Number of participants with readmission(s)
– Number of participants with failure to resolve obstruction
– Pain scores on the Visual Analog Scale
– Length of epidural catheter analgesia (days)
– Total need of opioids in milligrams
– Length of sick leave (days)
– Conversion rate (laparoscopic group)

Tertiary endpoints
– Number of participants who develop ventral hernia
– Number of patients with recurrent adhesive small bowel obstruction

Collected parameters in Case Report Form.

Sample size calculation
We have analyzed laparoscopic (n=12) and open (n=16) small bowel adhesiolysis operations performed in Helsinki University hospital 2010-2012. Patients who were operated laparoscopically, the mean hospital treatment time was 4,75 days and by open surgery 9,87 days. The mean standard deviation of treatment times were 3,65 days in laparoscopy group and 7,16 days in open surgery group. We estimated the standard deviation to be 3.75 days in laparoscopic group and 5 days in open surgery group. Sample size is calculated to be able to demonstrate 2.5 days difference in the post-operative length of stay. 102 patients are needed to achieve 80% power with a significance level of 0.05.

Schedule
The study is planned to start in summer 2013. The interim analysis will be done after recruitment of 51 patients. The trial is continued if there is no statistically significant difference between treatment groups in primary endpoint. However, if it is estimated that statistically significant difference is not possible to achieve with 102 trial patients, the study is ended. In other situations, the trial will end after recruitment and randomization of 102 patients. It is estimated that the recruitment will take 5 years, and the follow-up period is until 2038.

Registration
This trial will be registered at ClinicalTrials.gov

Privacy
Patient identification details are collected to two separate files. In one file is collected all patients who have adhesive small bowel obstruction in CT scan. To other file is collected those patients who are recruited to the trial. The data is stored and analyzed without identification details. Patients get randomization number which is stored with identification details to separate file. The data is stored in locked space and electronic data is stored in Helsinki university hospital’s computer which is protected by password.

References

1. Vettoretto N, Carrara A, Corradi A, De Vivo G, Lazzaro L, Ricciardelli L, et al. Laparoscopic adhesiolysis: consensus conference guidelines. Colorectal Disease. 2012 Mar 30;14(5):e208–15.

2. Barmparas G, Branco BC, Schnüriger B, Lam L, Inaba K, Demetriades D. The Incidence and Risk Factors of Post-Laparotomy Adhesive Small Bowel Obstruction. J Gastrointest Surg. 2010 Mar 30;14(10):1619–28.

3. Abbas S BIPB. Oral water soluble contrast for the management of adhesive small bowel obstruction. 2010 Jan 1;:1–24.

4. Branco BC, Barmparas G, Schnüriger B, Inaba K, Chan LS, Demetriades D. Systematic review and meta-analysis of the diagnostic and therapeutic role of water-soluble contrast agent in adhesive small bowel obstruction. Br J Surg. 2010 Mar 4;97(4):470–8.

5. Clotteau JE, Premont M. [Occlusion by adhesions treated by celioscopic section]. Presse Med. 1990 Jun 23;19(25):1196.

6. Li MZ, Lian L, Xiao L, Wu W, He Y, Song X. Laparoscopic versus open adhesiolysis in patients with adhesive small bowel obstruction: a systematic review and meta-analysis. AJS. Elsevier Inc; 2012 Jul 11;:1–8.

7. O'Connor DB, Winter DC. The role of laparoscopy in the management of acute small-bowel obstruction: a review of over 2,000 cases. Surg Endosc. 2011 Sep 5;26(1):12–7.

Research protocol version 1.2

Laparoscopic versus open adhesiolysis for small
bowel obstruction - a multicenter, prospective,
randomized, controlled trial

Ville Sallinen, Paulina Salminen, Eija Haukijärvi, Imre Ilves, Juha Saarnio, Vesa Koivukangas, Jyrki Kössi, Mikael Victorzon, Heidi Wikström, Salomone Di Saverio, Fausto Catena, Luca Ansaloni, Ari Leppäniemi, Panu Mentula

V1.2. 2.2.2015

Background

Bowel obstruction is one of the most common emergency surgical condition. Obstructive site is at 80% of the cases in small bowel. Adhesions are most common reason for small bowel obstruction (1,2). Most of the small bowel obstructions can be treated with non-operative treatment (3,4). Significant portion of the obstruction still need surgical intervention. Currently, the standard is to do explorative laparotomy. Commonly, the reason for obstruction is one adhesion and treatment is simple: to cut the band causing the adhesion. Laparoscopic approach seems ideal for such a procedure, preventing the morbidity of a laparotomy incision. First publications describing laparoscopic adhesiolysis in SBO are from the 1990's (5). Since then, several retrospective series comparing open and laparoscopic approach have been published. A recent meta-analysis pooled patients from four studies, including a total of 334 patients. Meta-analysis showed that patients treated by the laparoscopic approach had less complications, and faster return of bowel function (6). A recent consensus conference guideline recommends laparoscopic approach for selected patients (1). However, the scientific background of the recommendation is week, as there are no randomized or prospective studies of this subject. One of the drawbacks of laparoscopic approach is a concern for iatrogenic bowel perforation. In one report, the rate of bowel lesion in laparoscopic adhesiolysis was 6.6%, and only 84% were detected during the operation (7). In addition, there are no studies comparing the recurrence of small bowel obstructions between these two operative methods in long term follow-up.

Hypothesis

Laparoscopic adhesiolysis is suitable for treatment of acute small bowel obstruction and it shortens the hospital-stay time without increase in morbidity.

Methods

Centers

Helsinki university hospital
Turku university hospital
Tampere university hospital
Oulu university hospital
Kuopio university hospital
Päijät-Häme central hospital
Vaasa central hospital
Peijas hospital
Papa Giovanni XXIII Hospital, Bergamo, Italia
Maggiore Hospital Bologna, Bologna, Italia
Parma University Hospital, Parma, Italia

Patient evaluation and selection

Inclusion criteria

– All patients with clinical and computed tomography-diagnosed adhesive small bowel obstruction
– Obstruction is not relieved by conservative methods (nasogastric tube, nil per os) including Gastrografin® is not passed to colon within 8 hours (48-hour conservative treatment without Gastrografin® is allowed if Gastrografin® is contraindicated (e.g. allergy) or not available).

Exclusion criteria:

– Strong suspicion of strangulation or clinical peritonitis requiring an urgent operative intervention
– Previously confirmed or strongly suspected peritoneal carcinosis
– Previously confirmed wide diffuse adhesions of the abdominal cavity
– Previous open surgery for endometriosis
– Previous generalized peritonitis (not including local peritonitis such as appendicitis)
– Active abdominal malignancy or remission of less than 10 years' duration
– Previous radiotherapy of the abdominal region
– Previous obesity surgery
– 3 or more earlier open abdominal operations (not including caesarean section(s))
– Suspicion of other cause for obstruction than adhesions in CT-scan
– Recent abdominal operation (within 30 days)
– Previous laparotomy for aorta or iliac vessels
– Crohn's disease
– Anesthesiological contraindication for laparoscopy
– No informed consent
– Age less than 18 years or over 95 years
– Pregnancy
– Patient living in institutionalized care (such as health centre ward), not including retirement homes
– A hospital stay of more than one week prior to surgical consultation

Randomization procedure

Patients are randomly allocated (1:1) to either laparoscopic or open surgery. Randomization is done using block randomization with randomly varying block size (2–6) stratified by each study center. Cards with participants' randomization number and randomization group are sealed within numbered envelopes. Randomization and sealing within envelopes is done at the main research center (Helsinki) and letters are sent to each participating center at the beginning of the trial. The envelope is opened only after patient fulfills inclusion criteria, none of the exclusion criteria are met, and patient has agreed to participate in the study and has given a written consent. Envelopes are opened in numerical order. Operation is scheduled after randomization.

Recommendation for non-operative treatment before randomization

At emergency department, the nasogastric tube is set and blood samples (complete blood count, electrolytes, creatinine, CRP, liver enzymes, lactate, d-dimer) and abdominal CT ordered. Abdominal CT is done with IV contrast if possible. PO contrast is not used. Gastrografin passage is done after abdominal CT, but earliest 12 hours after nasogastric tube insertion. If po contrast does not pass to colon in 8 hours and patient meets the inclusion criteria and has no exclusion criteria, the patient is asked for study participation.

Intervention

Pre- and perioperative treatment

Fluid balance and electrolyte disturbances are corrected. Prophylactic cefuroxime 1500 mg and metronidazole 500 mg are administered intravenously just before the incision. An epidural catheter may be placed if recommended by the anesthesiologist. A nasogastric tube is inserted**.**

Laparoscopic technique

The first port is inserted using open approach or by using an optic port. Subsequent ports are inserted under direct vision. The location of the ports is left to the surgeon's discretion. The abdominal cavity is inspected and the caecum located and identified. Laparoscopic forceps are used to examine the small bowel starting from the terminal ileum until the transition site is identified. Dilated small bowel loops are not grasped but can be mobilized by grasping the mesenterium. Once the transition site is identified, the obstructing adhesions are divided, and the bowel is inspected for vitality. Ports are removed under vision, and possible bleeding is primarily controlled by ligatures. The fascial holes of ports over 5 mm are closed. A nasogastric tube is left in place.

Open surgical technique

A midline incision is made, and the abdominal cavity is inspected. The small bowel is examined until the transition site is located. Adhesions causing obstruction are divided. Excess fluid within small bowel is pushed into the stomach and the stomach is emptied using a nasogastric tube. The fascia is closed using continuous or interrupted sutures at surgeon's discretion.

Postoperative treatment

The nasogastric tube is kept in place until the secretion is less than 500 ml per 8 hours. After the removal of the nasogastric tube, the patient can drink up to 200 ml per 6 hours. If no nausea develops, patient may drink freely. Proton pump inhibitors are used for the length of the hospital stay. Thrombosis prophylaxis is commenced 6 hours after surgery, if there is no suspicion of postoperative hemorrhage. Ibuprofen, paracetamol, tramadol, and oxycodone can be used for pain. Pain is evaluated using a visual analogue scale daily and before administering painkillers.

Criteria for discharge

– Passage of stool
– The patient tolerates per oral nutrition
– Sufficient pain relieve is achieved with ibuprofen,
paracetamol, and/or tramadol.

Unresolving obstruction after surgery

If the obstruction if not resolved despite surgical treatment, the patient can undergo radiological imaging studies and/or surgical exploration (open or laparoscopic) at the discretion of the surgeon.

Follow-up

Patients of working age are given sick leave. The length of sick leave is at the discretion of treating physician, who is taking into consideration the patient's age and type of work (physical or desk job). A follow-up call is scheduled within 30 days, and return to work, possible late complications and readmissions are registered. Follow-up questionnaires are sent 1, 5 and 10 years after the randomization, and in case of no response, patients are contacted by telephone. Information about possible hernias and recurrent bowel obstructions are solicited.

Surgeons

The same surgeons perform both open and laparoscopic operations. All participating surgeons must have solid experience and skills of complex laparoscopic procedures, and need to have performed at least two laparoscopic adhesiolysis for small bowel obstruction before operating on patients participating in the trial.

Primary endpoint

– Length of post-operative hospital stay (days)

Secondary endpoints

– Passage of stool (post-operative days)
– Commencement of enteral nutrition (post-operative
days)
– 30-day mortality
– Complications (all causes), graded by Clavien-Dindo classification
– Number of participants with iatrogenic small bowel lesions
– Number of participants with readmission(s)
– Number of participants with failure to resolve obstruction
– Pain scores on the Visual Analog Scale
– Length of epidural catheter analgesia (days)
– Total need of opioids in milligrams

– Length of sick leave (days)
– Conversion rate (laparoscopic group)

Tertiary endpoints

– Number of participants who develop ventral hernia
– Number of patients with recurrent adhesive small bowel obstruction

Collected parameters in Case Report Form.

Sample size calculation

We have analyzed laparoscopic (n=12) and open (n=16) small bowel adhesiolysis operations performed in Helsinki University hospital 2010-2012. Patients who were operated laparoscopically, the mean hospital treatment time was 4,75 days and by open surgery 9,87 days. The mean standard deviation of treatment times were 3,65 days in laparoscopy group and 7,16 days in open surgery group. We estimated the standard deviation to be 3.75 days in laparoscopic group and 5 days in open surgery group. Sample size is calculated to be able to demonstrate 2.5 days difference in the post-operative length of stay. 102 patients are needed to achieve 80% power with a significance level of 0.05.

Schedule

The study is planned to start in summer 2013. The interim analysis will be done after recruitment of 51 patients. The trial is continued if there is no statistically significant difference between treatment groups in primary endpoint. However, if it is estimated that statistically significant difference is not possible to achieve with 102 trial patients, the study is ended. In other situations, the trial will end after recruitment and randomization of 102 patients. It is estimated that the recruitment will take 5 years, and the follow-up period is until 2038.

Registration

This trial will be registered at ClinicalTrials.gov

Privacy

Patient identification details are collected to two separate files. In one file is collected all patients who have adhesive small bowel obstruction in CT scan. To other file is collected those patients who are recruited to the trial. The data is stored and analyzed without identification details. Patients get randomization number which is stored with identification details to separate file. The data is stored in locked space and electronic data is stored in Helsinki university hospital's computer which is protected by password.

References

1. Vettoretto N, Carrara A, Corradi A, De Vivo G, Lazzaro L, Ricciardelli L, et al. Laparoscopic adhesiolysis: consensus conference guidelines. Colorectal Disease. 2012 Mar 30;14(5):e208–15.

2. Barmparas G, Branco BC, Schnüriger B, Lam L, Inaba K, Demetriades D. The Incidence and Risk Factors of Post-Laparotomy Adhesive Small Bowel Obstruction. J Gastrointest Surg. 2010 Mar 30;14(10):1619–28.

3. Abbas S BIPB. Oral water soluble contrast for the management of adhesive small bowel obstruction. 2010 Jan 1;:1–24.

4. Branco BC, Barmparas G, Schnüriger B, Inaba K, Chan LS, Demetriades D. Systematic review and meta-analysis of the diagnostic and therapeutic role of water-soluble contrast agent in adhesive small bowel obstruction. Br J Surg. 2010 Mar 4;97(4):470–8.

5. Clotteau JE, Premont M. [Occlusion by adhesions treated by celioscopic section]. Presse Med. 1990 Jun 23;19(25):1196.

6. Li MZ, Lian L, Xiao L, Wu W, He Y, Song X. Laparoscopic versus open adhesiolysis in patients with adhesive small bowel obstruction: a systematic review and meta-analysis. AJS. Elsevier Inc; 2012 Jul 11;:1–8.

7. O'Connor DB, Winter DC. The role of laparoscopy in the management of acute small-bowel obstruction: a review of over 2,000 cases. Surg Endosc. 2011 Sep 5;26(1):12–7.

Research protocol version 1.3

Laparoscopic versus open adhesiolysis for small bowel obstruction - a multicenter, prospective, randomized, controlled trial

Ville Sallinen, Salomone DiSaverio, Eija Haukijärvi, Risto Juusela, Vesa Koivukangas, Heidi Wikström, Fausto Catena, Panu Räty, Arianna Birindelli, Ari Leppäniemi, Panu Mentula

V1.3 (2.10.2018)

Background

Bowel obstruction is one of the most common emergency surgical condition. Obstructive site is at 80% of the cases in small bowel. Adhesions are most common reason for small bowel obstruction (1,2). Most of the small bowel obstructions can be treated with non-operative treatment (3,4). Significant portion of the obstruction still need surgical intervention. Currently, the standard is to do explorative laparotomy. Commonly, the reason for obstruction is one adhesion and treatment is simple: to cut the band causing the adhesion. Laparoscopic approach seems ideal for such a procedure, preventing the morbidity of a laparotomy incision. First publications describing laparoscopic adhesiolysis in SBO are from the 1990's (5). Since then, several retrospective series comparing open and laparoscopic approach have been published. A recent meta-analysis pooled patients from four studies, including a total of 334 patients. Meta-analysis showed that patients treated by the laparoscopic approach had less complications, and faster return of bowel function (6). A recent consensus conference guideline recommends laparoscopic approach for selected patients (1). However, the scientific background of the recommendation is week, as there are no randomized or prospective studies of this subject. One of the drawbacks of laparoscopic approach is a concern for iatrogenic bowel perforation. In one report, the rate of bowel lesion in laparoscopic adhesiolysis was 6.6%, and only 84% were detected during the operation (7). In addition, there are no studies comparing the recurrence of small bowel obstructions between these two operative methods in long term follow-up.

Hypothesis

Laparoscopic adhesiolysis is suitable for treatment of acute small bowel obstruction and it shortens the hospital-stay time without increase in morbidity.

Methods

Centers

Helsinki university hospital
Turku university hospital
Tampere university hospital
Oulu university hospital
Kuopio university hospital
Päijät-Häme central hospital
Vaasa central hospital
Peijas hospital
Papa Giovanni XXIII Hospital, Bergamo, Italia
Maggiore Hospital Bologna, Bologna, Italia
Parma University Hospital, Parma, Italia

Patient evaluation and selection

Inclusion criteria

– All patients with clinical and computed tomography-diagnosed adhesive small bowel obstruction
– Obstruction is not relieved by conservative methods (nasogastric tube, nil per os) including Gastrografin® is not passed to colon within 8 hours (48-hour conservative treatment without Gastrografin® is allowed if Gastrografin® is contraindicated (e.g. allergy) or not available).

Exclusion criteria:

– Strong suspicion of strangulation or clinical peritonitis requiring an urgent operative intervention
– Previously confirmed or strongly suspected peritoneal carcinosis
– Previously confirmed wide diffuse adhesions of the abdominal cavity
– Previous open surgery for endometriosis
– Previous generalized peritonitis (not including local peritonitis such as appendicitis)
– Active abdominal malignancy or remission of less than 10 years' duration
– Previous radiotherapy of the abdominal region
– Previous obesity surgery
– 3 or more earlier open abdominal operations (not including caesarean section(s))
– Suspicion of other cause for obstruction than adhesions in CT-scan
– Recent abdominal operation (within 30 days)
– Previous laparotomy for aorta or iliac vessels
– Crohn's disease
– Anesthesiological contraindication for laparoscopy
– No informed consent
– Age less than 18 years or over 95 years
– Pregnancy
– Patient living in institutionalized care (such as health centre ward), not including retirement homes
– A hospital stay of more than one week prior to surgical consultation

Randomization procedure

Patients are randomly allocated (1:1) to either laparoscopic or open surgery. Randomization is done using block randomization with randomly varying block size (2–6) stratified by each study center. Cards with participants' randomization number and randomization group are sealed within numbered envelopes. Randomization and sealing within envelopes is done at the main research center (Helsinki) and letters are sent to each participating center at the beginning of the trial. The envelope is opened only after patient fulfills inclusion criteria, none of the exclusion criteria are met, and patient has agreed to participate in the study and has given a written consent. Envelopes are opened in numerical order. Operation is scheduled after randomization.

Recommendation for non-operative treatment before randomization

At emergency department, the nasogastric tube is set and blood samples (complete blood count, electrolytes, creatinine, CRP, liver enzymes, lactate, d-dimer) and abdominal CT ordered. Abdominal CT is done with IV contrast if possible. PO contrast is not used. Gastrografin passage is done after abdominal CT, but earliest 12 hours after nasogastric tube insertion. If po contrast does not pass to colon in 8 hours and patient meets the inclusion criteria and has no exclusion criteria, the patient is asked for study participation.

Intervention

Pre- and perioperative treatment

Fluid balance and electrolyte disturbances are corrected. Prophylactic cefuroxime 1500 mg and metronidazole 500 mg are administered intravenously just before the incision. An epidural catheter may be placed if recommended by the anesthesiologist. A nasogastric tube is inserted**.**

Laparoscopic technique

The first port is inserted using open approach or by using an optic port. Subsequent ports are inserted under direct vision. The location of the ports is left to the surgeon's discretion. The abdominal cavity is inspected and the caecum located and identified. Laparoscopic forceps are used to examine the small bowel starting from the terminal ileum until the transition site is identified. Dilated small bowel loops are not grasped but can be mobilized by grasping the mesenterium. Once the transition site is identified, the obstructing adhesions are divided, and the bowel is inspected for vitality. Ports are removed under vision, and possible bleeding is primarily controlled by ligatures. The fascial holes of ports over 5 mm are closed. A nasogastric tube is left in place.

Open surgical technique

A midline incision is made, and the abdominal cavity is inspected. The small bowel is examined until the transition site is located. Adhesions causing obstruction are divided. Excess fluid within small bowel is pushed into the stomach and the stomach is emptied using a nasogastric tube. The fascia is closed using continuous or interrupted sutures at surgeon's discretion.

Postoperative treatment

The nasogastric tube is kept in place until the secretion is less than 500 ml per 8 hours. After the removal of the nasogastric tube, the patient can drink up to 200 ml per 6 hours. If no nausea develops, patient may drink freely. Proton pump inhibitors are used for the length of the hospital stay. Thrombosis prophylaxis is commenced 6 hours after surgery, if there is no suspicion of postoperative hemorrhage. Ibuprofen, paracetamol, tramadol, and oxycodone can be used for pain. Pain is evaluated using a visual analogue scale daily and before administering painkillers.

Criteria for discharge
– Passage of stool
– The patient tolerates per oral nutrition
– Sufficient pain relieve is achieved with ibuprofen,
paracetamol, and/or tramadol.

Unresolving obstruction after surgery
If the obstruction if not resolved despite surgical treatment, the patient can undergo radiological imaging studies and/or surgical exploration (open or laparoscopic) at the discretion of the surgeon.

Follow-up
Patients of working age are given sick leave. The length of sick leave is at the discretion of treating physician, who is taking into consideration the patient's age and type of work (physical or desk job). A follow-up call is scheduled within 30 days, and return to work, possible late complications and readmissions are registered. Follow-up questionnaires are sent 1, 5 and 10 years after the randomization, and in case of no response, patients are contacted by telephone. Information about possible hernias and recurrent bowel obstructions is solicited. At five and ten years of follow-up, the patients are also sent Gastrointestinal Quality of Life Index (GIQLI) and SF-36 questionnaires.

Surgeons

The same surgeons perform both open and laparoscopic operations. All participating surgeons must have solid experience and skills of complex laparoscopic procedures, and need to have performed at least two laparoscopic adhesiolysis for small bowel obstruction before operating on patients participating in the trial.

Primary endpoint

– Length of post-operative hospital stay (days)

Secondary endpoints

– Passage of stool (post-operative days)
– Commencement of enteral nutrition (post-operative days)
– 30-day mortality
– Complications (all causes), graded by Clavien-Dindo classification
– Number of participants with iatrogenic small bowel lesions
– Number of participants with readmission(s)
– Number of participant with failure to resolve obstruction
– Pain scores on the Visual Analog Scale
– Length of epidural catheter analgesia (days)
– Total need of opioids in milligrams

– Length of sick leave (days)
– Conversion rate (laparoscopic group)

Tertiary endpoints

– Number of participants who develop ventral hernia
– Number of patients with recurrent adhesive small bowel obstruction

Collected parameters in Case Report Form.

Sample size calculation

We have analyzed laparoscopic (n=12) and open (n=16) small bowel adhesiolysis operations performed in Helsinki University hospital 2010-2012. Patients who were operated laparoscopically, the mean hospital treatment time was 4,75 days and by open surgery 9,87 days. The mean standard deviation of treatment times were 3,65 days in laparoscopy group and 7,16 days in open surgery group. We estimated the standard deviation to be 3.75 days in laparoscopic group and 5 days in open surgery group. Sample size is calculated to be able to demonstrate 2.5 days difference in the post-operative length of stay. 102 patients are needed to achieve 80% power with a significance level of 0.05.

Schedule

The study is planned to start in summer 2013. The interim analysis will be done after recruitment of 51 patients. The trial is continued if there is no statistically significant difference between treatment groups in primary endpoint. However, if it is estimated that statistically significant difference is not possible to achieve with 102 trial patients, the study is ended. In other situations, the trial will end after recruitment and randomization of 102 patients. It is estimated that the recruitment will take 5 years, and the follow-up period is until 2038.

Registration

This trial will be registered at ClinicalTrials.gov

Privacy

Patient identification details are collected to two separate files. In one file is collected all patients who have adhesive small bowel obstruction in CT scan. To other file is collected those patients who are recruited to the trial. The data is stored and analyzed without identification details. Patients get randomization number which is stored with identification details to separate file. The data is stored in locked space and electronic data is stored in Helsinki university hospital's computer which is protected by password.

References

1. Vettoretto N, Carrara A, Corradi A, De Vivo G, Lazzaro L, Ricciardelli L, et al. Laparoscopic adhesiolysis: consensus conference guidelines. Colorectal Disease. 2012 Mar 30;14(5):e208–15.

2. Barmparas G, Branco BC, Schnüriger B, Lam L, Inaba K, Demetriades D. The Incidence and Risk Factors of Post-Laparotomy Adhesive Small Bowel Obstruction. J Gastrointest Surg. 2010 Mar 30;14(10):1619–28.

3. Abbas S BIPB. Oral water soluble contrast for the management of adhesive small bowel obstruction. 2010 Jan 1;:1–24.

4. Branco BC, Barmparas G, Schnüriger B, Inaba K, Chan LS, Demetriades D. Systematic review and meta-analysis of the diagnostic and therapeutic role of water-soluble contrast agent in adhesive small bowel obstruction. Br J Surg. 2010 Mar 4;97(4):470–8.

5. Clotteau JE, Premont M. [Occlusion by adhesions treated by celioscopic section]. Presse Med. 1990 Jun 23;19(25):1196.

6. Li MZ, Lian L, Xiao L, Wu W, He Y, Song X. Laparoscopic versus open adhesiolysis in patients with adhesive small bowel obstruction: a systematic review and meta-analysis. AJS. Elsevier Inc; 2012 Jul 11;:1–8.

7. O'Connor DB, Winter DC. The role of laparoscopy in the management of acute small-bowel obstruction: a review of over 2,000 cases. Surg Endosc. 2011 Sep 5;26(1):12–7.
