# Supplementary material for: Long-Term Outcomes After Laparoscopic vs Open Adhesiolysis for Small Bowel Obstruction: The LASSO Randomized Clinical Trial
Source: JAMA Surg. 2026 Feb 18;161(4):381–8. doi: 10.1001/jamasurg.2025.6726 (PMC12917750; doi:10.1001/jamasurg.2025.6726)
Supplement: Supplement 2. — Statistical Analysis Plan. [file jamasurg-e256726-s002.pdf]

Statistical analysis plan

Continuous outcomes (QIGLI and SF-36 scores) that are not normally distributed are analyzed using Mann-Whitney U-test (after multiple imputation with median p-rule -method) and effect size reported as $r = Z/\sqrt{N}$. Multiple imputation (linear regression method) with ten imputed data sets is used only in GIQLI questionnaire analysis, if at least 75% of the questionnaire was filled out. The missing data are assumed to be missing at random. Categorical outcomes are analyzed using Fisher's exact test, $\chi 2$ test and effect size is reported as odds ratio (OR) with 95% CI. All analyses are performed with SPSS version 29 (IBM, Armonk, NY).

Main analyses are carried out according to intention-to-treat principle. In addition, post hoc per-protocol analyses are carried out where patients who were randomized to laparoscopic adhesiolysis, but underwent conversion to open surgery, are excluded from analyses. Potential effects of attrition are examined by comparing original baseline demographics of patients who responded to one-year follow up, five-year QOL questionnaire and patients who were randomized to initial study.
