# Supplementary material for: Long-Term Outcomes After Laparoscopic vs Open Adhesiolysis for Small Bowel Obstruction: The LASSO Randomized Clinical Trial
Source: JAMA Surg. 2026 Feb 18;161(4):381–8. doi: 10.1001/jamasurg.2025.6726 (PMC12917750; doi:10.1001/jamasurg.2025.6726)
Supplement: Supplement 3. — eTable 1. Baseline Demographics of Patients Who Were Included to Initial Study and Patients Who Were in 1-Year Analysis eTable 2. One-Year Analysis of Outcomes for Patients Randomized to Laparoscopic Approach but Converted to Open Surgery eTable 3. Five-Year Analysis of Outcomes for Patients Randomized to Laparoscopic Approach but Converted to Open Surgery eTable 4. Baseline Demographics of Patients Who Responded to 5-Year QOL (Quality of Life) Questionnaire eTable 5. Baseline Demographics of Patients Who Were Included to Initial Study and Patients Who Were in 5-Year QOL Analysis [file jamasurg-e256726-s003.pdf]

Supplementary Online Content

Räty P, Mentula P, Haukijärvi E, et al. Long-term outcomes after laparoscopic vs open adhesiolysis for small bowel obstruction: the LASSO randomized clinical trial. *JAMA Surg*. Published online February 18, 2026. doi:10.1001/jamasurg.2025.6726

eTable 1. Baseline Demographics of Patients Who Were Included to Initial Study and Patients Who Were in 1-Year Analysis

eTable 2. One-Year Analysis of Outcomes for Patients Randomized to Laparoscopic Approach but Converted to Open Surgery

eTable 3. Five-Year Analysis of Outcomes for Patients Randomized to Laparoscopic Approach but Converted to Open Surgery

eTable 4. Baseline Demographics of Patients Who Responded to 5-Year QOL (Quality of Life) Questionnaire

eTable 5. Baseline Demographics of Patients Who Were Included to Initial Study and Patients Who Were in 5-Year QOL Analysis

This supplementary material has been provided by the authors to give readers additional information about their work.

© 2026 Räty P et al. *JAMA Surgery*.

	Patients in one-year analysis	Patients included to initial study
Median age, years (IQR)	73 (59-81)	74 (60-83)
Sex, male, no. (%)	32 (36)	35 (35)
Body-mass index, kg/m2 (IQR)	23.2 (20.9-26.1)	23.5 (20.9-26.4)
ASA physical status, no. (%)		
1	7 (8)	7 (7)
2	26 (30)	28 (28)
3	39 (44)	43 (43)
4	16 (18)	22 (22)
Comorbidities, no. (%)		
Myocardial infarction	6 (7)	8 (8)
Congestive heart failure	3 (3)	5 (5)
Coronary disease	9 (10)	10 (10)
Hypertension	32 (36)	39 (39)
Peripheral vascular disease	3 (3)	3 (3)
Cerebro vascular disease	4 (5)	6 (6)
Hemiplegia	0	0
COPD	11 (13)	14 (14)
Connective tissue disease	1 (1)	1 (1)
Liver disease		
Mild	1 (1)	2 (2)
Moderate or severe	0	0
Peptic ulcer disease	3 (3)	3 (3)
Diabetes		
Without complications	7 (8)	9 (9)
With complication	0	1 (1)
Kidney disease (moderate or severe)	1 (1)	2 (2)
Solid tumor	2 (2)	3 (3)
Metastatic malignancy	1 (1)	3 (3)
Leukemia	0	0
Lymphoma	1 (1)	1 (1)
AIDS	0	0
No comorbidities	24 (27)	24 (24)
Number of previously conservatively managed ileus		
0	79 (92)	88 (90)
1	6 (7)	9 (9)
2	1 (1)	1 (1)
Number of earlier operations		
0	9 (10)	10 (10)
1	45 (51)	49 (49)
2	30 (34)	36 (36)

© 2026 Räty P et al. *JAMA Surgery*.

3	4 (5)	5 (5)

eTable 1. Baseline demographics of patients who were included to initial study and patients who were in one-year analysis. There were no differences between the groups.

© 2026 Räty P et al. *JAMA Surgery*.

	Laparoscopy n=32	Conversion n=12	Open surgery n=44	p
**SBO within one year, No. (%)**	2 (6)	0	1 (2)	0.72[a]
**Nonoperatively treated SBO within one year, No. (%)**	2 (6)	0	1 (2)	0.72[a]
**Operatively treated SBO within one year, No. (%)**	0	0	0	NA
**Incisional hernia within one year, No. (%)**	0	2 (17)	2 (5)	0.09[a]

eTable 2. One-year analysis of outcomes for patients randomized to laparoscopic approach but converted to open surgery.
[a]Fisher's exact test -test.

© 2026 Räty P et al. *JAMA Surgery*.

	Laparoscopy n=23	Conversion n=9	Open surgery n=31	p
**SBO within five years, No. (%)**	3 (13)	1 (11)	3 (10)	1.00[a]
**Nonoperatively treated SBO within five years, No. (%)**	3 (13)	1 (11)	2 (7)	0.62[a]
**Operatively treated SBO within five years, No. (%)**	1 (4)	1 (11)	2 (7)	0.63[a]
**Incisional hernia within five years#, No. (%)**	0	2 (22)	2 (6)	0.09[a]

eTable 3. Five-year analysis of outcomes for patients randomized to laparoscopic approach but converted to open surgery
[a]Fisher's exact test -test. #open surgery group n=33

© 2026 Räty P et al. *JAMA Surgery*.

	Open surgery (n=32)	Laparoscopy (n=31)
Median age, years (IQR)	67.5 (56.3-76.3)	68 (54-79)
Sex, male, no. (%)	11 (34)	9 (29)
Body-mass index, kg/m2 (IQR)	23.4 (20.6-25.0)	23.9 (21.4-29.4)
ASA physical status, no. (%)		
1	5 (16)	2 (7)
2	10 (31)	12 (39)
3	13 (41)	14 (45)
4	4 (13)	3 (10)
Comorbidities, no. (%)		
Myocardial infarction	1 (3)	1 (3)
Congestive heart failure	1 (3)	0
Coronary disease	3 (9)	2 (7)
Hypertension	8 (25)	13 (42)
Peripheral vascular disease	0	2 (7)
Cerebro vascular disease	2 (6)	2 (6)
Hemiplegia	0	0
COPD	1 (3)	2 (7)
Connective tissue disease	0	1 (3)
Liver disease		
Mild	0	0
Moderate or severe	0	0
Peptic ulcer disease	3 (9)	0
Diabetes		
Without complications	3 (9)	3 (10)
With complication	0	0
Kidney disease (moderate or severe)	0	0
Solid tumor	1 (3)	1 (3)
Metastatic malignancy	0	0
Leukemia	0	0
Lymphoma	0	0
AIDS	0	0
No comorbidities	11 (34)	10 (32)
Number of previously conservatively managed ileus		
0	28 (88)	28 (90)
1	3 (9)	3 (10)
2	1 (3)	0
Number of earlier operations		
0	2 (6)	5 (16)
1	20 (63)	13 (42)
2	9 (28)	13 (42)

© 2026 Räty P et al. *JAMA Surgery*.

3	1 (3)	0

eTable 4. Baseline demographics of patients who responded to five-year QOL (quality of life) questionnaire. There were no statistical differences between patients in different groups who responded to five-year QOL questionnaire.

© 2026 Räty P et al. *JAMA Surgery*.

	Patients who responded to 5-year QOL survey	Patients included to initial study
Median age, years (IQR)	68 (56-77)	74 (60-83)
Sex, male, no. (%)	20 (32)	35 (35)
Body-mass index, kg/m2 (IQR)	23.3 (20.8-26.4)	23.5 (20.9-26.4)
ASA physical status, no. (%)		
1	7 (11)	7 (7)
2	22 (35)	28 (28)
3	27 (43)	43 (43)
4	7 (11)	22 (22)
Comorbidities, no. (%)		
Myocardial infarction	2 (3)	8 (8)
Congestive heart failure	1 (2)	5 (5)
Coronary disease	5 (8)	10 (10)
Hypertension	21 (33)	39 (39)
Peripheral vascular disease	2 (3)	3 (3)
Cerebro vascular disease	4 (6)	6 (6)
Hemiplegia	0	0
COPD	3 (5)	14 (14)
Connective tissue disease	1 (2)	1 (1)
Liver disease		
Mild	0	2 (2)
Moderate or severe	0	0
Peptic ulcer disease	3 (5)	3 (3)
Diabetes		
Without complications	6 (10)	9 (9)
With complication	0	1 (1)
Kidney disease (moderate or severe)	0	2 (2)
Solid tumor	2 (3)	3 (3)
Metastatic malignancy	0	3 (3)
Leukemia	0	0
Lymphoma	0	1 (1)
AIDS	0	0
No comorbidities	21 (33)	24 (24)
Number of previously conservatively managed ileus		
0	56 (89)	88 (90)
1	6 (10)	9 (9)
2	1 (2)	1 (1)
Number of earlier operations		
0	7 (11)	10 (10)
1	33 (52)	49 (49)

© 2026 Räty P et al. *JAMA Surgery*.

2	22 (35)	36 (36)
3	1 (2)	5 (5)

eTable 5. Baseline demographics of patients who were included to initial study and patients who were in five-year QOL analysis. The only detected statistical difference was patient age (p=0.004, analyzed with Mann-Whitney U-test)

© 2026 Räty P et al. *JAMA Surgery*.
