# Supplementary material for: Long-Term Outcomes After Laparoscopic vs Open Adhesiolysis for Small Bowel Obstruction: The LASSO Randomized Clinical Trial
Source: JAMA Surg. 2026 Feb 18;161(4):381–8. doi: 10.1001/jamasurg.2025.6726 (PMC12917750; doi:10.1001/jamasurg.2025.6726)
Supplement: Supplement 4. — Data Sharing Statement. [file jamasurg-e256726-s004.pdf]

Data Sharing Statement

Räty. Long-Term Outcomes After Laparoscopic vs Open Adhesiolysis for Small Bowel Obstruction. *JAMA Surg*. Published February 18, 2026. doi:10.1001/jamasurg.2025.6726

Data
Additional Information: NCT01867528
Data available: No

Additional Information
Explanation for why data not available: Study permissions do not allow data sharing.
